# Robust Quantification of Affected Brain Volume from Computed Tomography Perfusion: A Hybrid Approach Combining Deep Learning and Singular Value Decomposition

**DOI:** 10.1007/s10278-025-01612-5

**Published:** 2025-08-27

**Authors:** Gi-Youn Kim, Hyeon Sik Yang, Jundong Hwang, Kijeong Lee, Jin Wook Choi, Woo Sang Jung, Regina Eun Young Kim, Donghyeon Kim, Minho Lee

**Affiliations:** 1 Research Institute, Neurophet Inc., 12F, 124, Teheran-ro, Gangnam-gu Seoul, Republic of Korea; 2 Department of Radiology, Ajou University School of Medicine, Suwon, Republic of Korea

**Keywords:** Computed tomography perfusion (CTP), Volumetric estimation, Hybrid model, Deep learning (DL), Vascular landmarks, Singular value decomposition (SVD)

## Abstract

Volumetric estimation of affected brain volumes using computed tomography perfusion (CTP) is crucial in the management of acute ischemic stroke (AIS) and relies on commercial software, which has limitations such as variations in results due to image quality. To predict affected brain volume accurately and robustly, we propose a hybrid approach that integrates singular value decomposition (SVD), deep learning (DL), and machine learning (ML) techniques. We included 449 CTP images of patients with AIS with manually annotated vessel landmarks provided by expert radiologists, collected between 2021 and 2023. We developed a CNN-based approach for predicting eight vascular landmarks from CTP images, integrating ML components. We then used SVD-related methods to generate perfusion maps and compared the results with those of the RapidAI software (RapidAI, Menlo Park, California). The proposed CNN model achieved an average Euclidean distance error of 4.63 $\pm$ 2.00 mm on the vessel localization. Without the ML components, compared to RapidAI, our method yielded concordance correlation coefficient (CCC) scores of 0.898 for estimating volumes with cerebral blood flow (CBF) < 30% and 0.715 for Tmax > 6 s. Using the ML method, it achieved CCC scores of 0.905 for CBF < 30% and 0.879 for Tmax > 6 s. For the data assessment, it achieved 0.8 accuracy. We developed a robust hybrid model combining DL and ML techniques for volumetric estimation of affected brain volumes using CTP in patients with AIS, demonstrating improved accuracy and robustness compared to existing commercial solutions.

## Introduction

In patients with acute ischemic stroke (AIS), predicting the brain volume affected by the index stroke plays a crucial role in determining the appropriate treatment strategy, especially for reperfusion therapy [1–3]. One of the primary imaging techniques used for this analysis is computed tomography perfusion (CTP), which provides dynamic blood flow images through rapid injection of contrast agents. Perfusion maps generated from CTP images provides indicators, such as cerebral blood flow (CBF), cerebral blood volume (CBV), and mean transit time (MTT), which are essential for accurately calculating the brain areas affected by the AIS. Extracting these indicators requires setting regions of interest (ROIs) to calculate the arterial input function (AIF) and venous output function (VOF) [4].

However, in clinical settings, neuroradiologists may not always be available to manually identify ROIs. Therefore, commercial software, such as RapidAI (RapidAI, Menlo Park, California), Olea Sphere (Olea Medical, La Ciotat, France), and Vitrea (Canon Medical Informatics, Minnetonka, Minnesota), have been used to automatically identify ROIs and generate perfusion maps [5]. Such software is widely adopted in the repetitive parts of stroke-related clinical workflows to simplify workflows for radiologists and other frontline physicians [6, 7]. Although these software solutions are clinically useful, they have been criticized for their lack of robustness. Some studies have pointed out that such software generates summary reports even when a given CTP image is not appropriate for use [8, 9].

Additionally, automated CTP image post-processing methods may still produce results despite poor input quality, requiring careful interpretation [10]. Despite these drawbacks, existing studies have focused more on accurate volume prediction than on detecting inadequate input data using deep learning (DL) techniques [11–13].

For instance, Varies et al. proposed PerfU-Net, an attention-mechanism-based U-Net that does not rely on commercial CTP software [12]. This model uses CTP image as input and achieved comparable volume segmentation performance to studies utilizing perfusion maps from commercial software combined with non-contrast CT scans. Bhurwani et al. developed a U-Net-based model that used perfusion maps composed of CBF, CBV, MTT, Tmax, and delay to segment the affected brain volumes [11]. Their results were closer to the actual volumes observed in diffusion-weighted imaging (DWI)/FLAIR than those obtained using traditional thresholding mechanisms from commercial software. Zhu et al. introduced ISP-Net, which uses CTP image data and perfusion maps as inputs and incorporates a multiscale atrous convolution (MSAC) block to extract contextual information [13]. Unlike previous studies, they additionally used perfusion maps generated by the GE CT Perfusion 4D software, achieving a volume-segmentation Dice score of 0.801. Amador et al. proposed a U-Net-based convolutional neural network (CNN) employing a temporal convolutional network using 4D CTP image as the input [14]. This study eliminated the traditional volume segmentation procedure via perfusion maps, and unlike other studies that used two-dimensional spatiotemporal (2D + T) data as input, they were able to use three-dimensional spatiotemporal (3D + T) data.

These studies still emphasized that the threshold criteria used by commercial software for predicting affected brain volumes vary by vendor, making cross-comparisons difficult and questioning their robustness [11–13]. Nonetheless, most of the studies did not focus on preventing the misleading predictions that affect clinical decisions. In the medical domain, robustness is essential for ensuring accurate and consistent analysis results, regardless of the input data quality [15].

Therefore, with advancements in AI technology that can improve the accuracy of volume prediction, there is a need to introduce new techniques that can enhance clinical completeness while retaining existing solutions. In this study, we propose a robust hybrid model that combines DL and machine learning (ML) to predict AIS-affected brain volumes. For vessel localization, we used DL. For validity checking, we used ML. For lesion quantification, we used block-circulant singular value decomposition (bcSVD). This approach addresses the limitations of commercial software in volume prediction accuracy and overcomes the challenges of existing deep-learning-based techniques in effectively assessing input data.

## Methods and Materials

### Dataset

Table 1 provides an overview of the collected dataset. The collected dataset included 449 CTP images, each accompanied by an ROI and analysis results from RapidAI software. DWI was also provided for a few data at the same time. Before the training and validation, the data was organized into several groups. From the entire dataset, 39 cases were identified where the RapidAI software generated warnings due to bad bolus timing; these cases were assigned to Group 1. Additionally, 42 cases where our proposed method indicated “invalid curve (warning)” were assigned to Group 2. We excluded six datasets obtained from a Toshiba scanner from the analysis.

**Table 1:** Overview of dataset

Data groups	Data type	Vendor	Train/val
Group 1 (8.6%, *N* = 39)	RapidAI warning cases	Siemens	Test 2
Group 2 (9.3%, *N* = 42)	Proposed method warning cases	Siemens	Test 2
Group 3 (80.6%, *N* = 362)	Standard dataset	Siemens	Train validation Test 1
Etc (1.3%, *N* = 6)	Exclude	Toshiba	-
Total (100%, *N* = 449)	-	-	-

The remaining 362 scans, excluding the cases in Groups 1 and 2 from the entire dataset, formed the Standard Dataset (Group 3). This dataset was randomly divided into training and test sets in an 80:20 ratio, 290 for training and 72 for Test 1, which aimed to demonstrate that the proposed method produces accurate perfusion maps and reliable AIS-affected brain volume estimations. Within the training, we randomly selected 10% as validation set during model train process. Furthermore, to assess the input data assessment ability of our method, we conducted Validation 2 using the combined data from Groups 1 and 2.

### CTP Acquisition

CTP images were acquired using Siemens scanners (Definition Edge, Definition Flash, and Force models) with the following scan parameters: tube voltage of 80/70 kVp (80 kVp for Definition Edge and Definition Flash, 70 kVp for Force), tube current of 200 mAs, single collimation width of 1.2 mm, and total collimation width of 38.4 mm (Definition Edge and Definition Flash) or 57.6 mm (Force). Thirty cycles were captured every 1.5 s during the arterial phase, resulting in a total acquisition time of 45 s. Images were reconstructed with a matrix size of 512 × 512 pixels and a slice thickness of 4 mm. The total z-axis coverage was approximately 9 cm.

### Labeling for ROI Localization

For each CTP image, the ROIs of AIF and VOF were initially annotated by trained annotators and subsequently reviewed and refined by two expert radiologists (22, 13 years of experience each). These ROIs served as the ground truth (GT) for the ROI localization model and served as AIF/VOF location. The analysis results from the RapidAI software were presented as volumes F where it represents the area of the brain with a relative cerebral blood flow (rCBF) < 30%, while Volume T corresponds to regions where the time-to-maximum (Tmax) exceeds 6 s. Volume values were stored as integers, accompanied by metadata on warnings generated by the RapidAI software. The characteristics of AIS-affected brain volume make it challenging to establish a precise ground truth, so we used the RapidAI software’s volume values as the ground truth and evaluate similarity accordingly [11, 16].

### Preprocessing

All scans were preprocessed before being utilized as input for the proposed method. The preprocessing steps included skull stripping, motion correction, resampling, resizing, maximum intensity projection (MIP), and z-score normalization. By applying 3D rigid registration, motion correction was applied to correct any shifts in position along the temporal axis, and the corrected image was then uniformly resampled to achieve a consistent slice thickness of 4 mm.

While MIP is typically applied along the time axis in 3D + T data—a common characteristic of CTP image—we aimed to locate the ROIs corresponding to the vessels. Therefore, we constructed the MIP data by subtracting the minimum intensity from the maximum intensity across the entire 3D image along the time axis. Subsequently, we performed resizing with cropping or padding to produce MIP data with dimensions of 512 × 512 × 27 voxels, to make all data shapes uniform and then utilize them as input for the DL model.

### Proposed Method

**Fig. 1:**
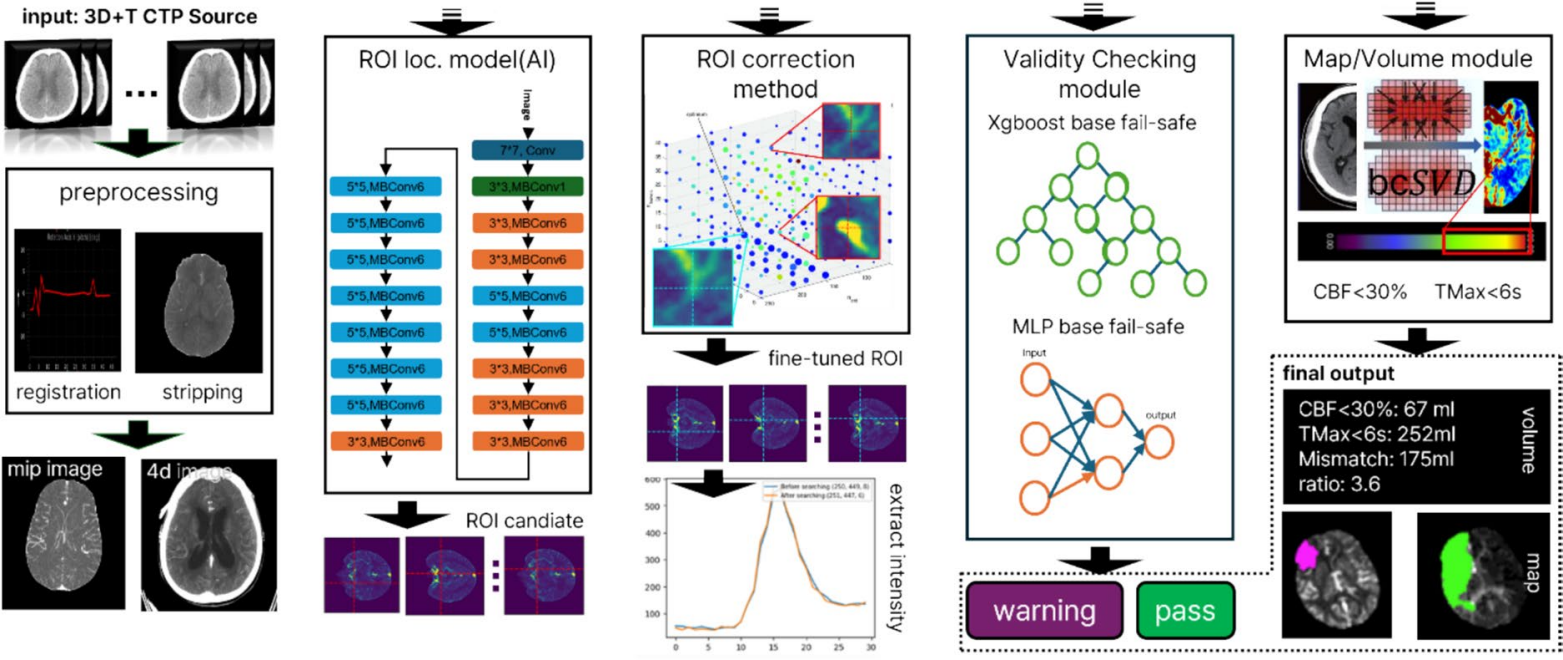
Overview of proposed method

Figure 1 presents an overview of the proposed method. In the localization model step, we developed a CNN to automatically identify the ROIs associated with the vessels in the CTP images. Based on the CNN’s predictions, we employed a simple grid-search algorithm to refine the selection of vessel locations near the predicted ROIs during the ROI correction step. To further ensure the validity of the extracted AIF/VOF curves, we applied ML techniques to assess whether these curves exhibited the characteristic shapes of true arterial and venous functions and belonged to arterial and venous functions in the validity-checking step. This process filtered out any atypical or erroneous curves. Using the refined AIF/VOF curves, we performed block-circulant singular value decomposition (bcSVD) to compute the final Volumes T and F.

#### Localization Model

**Fig. 2:**
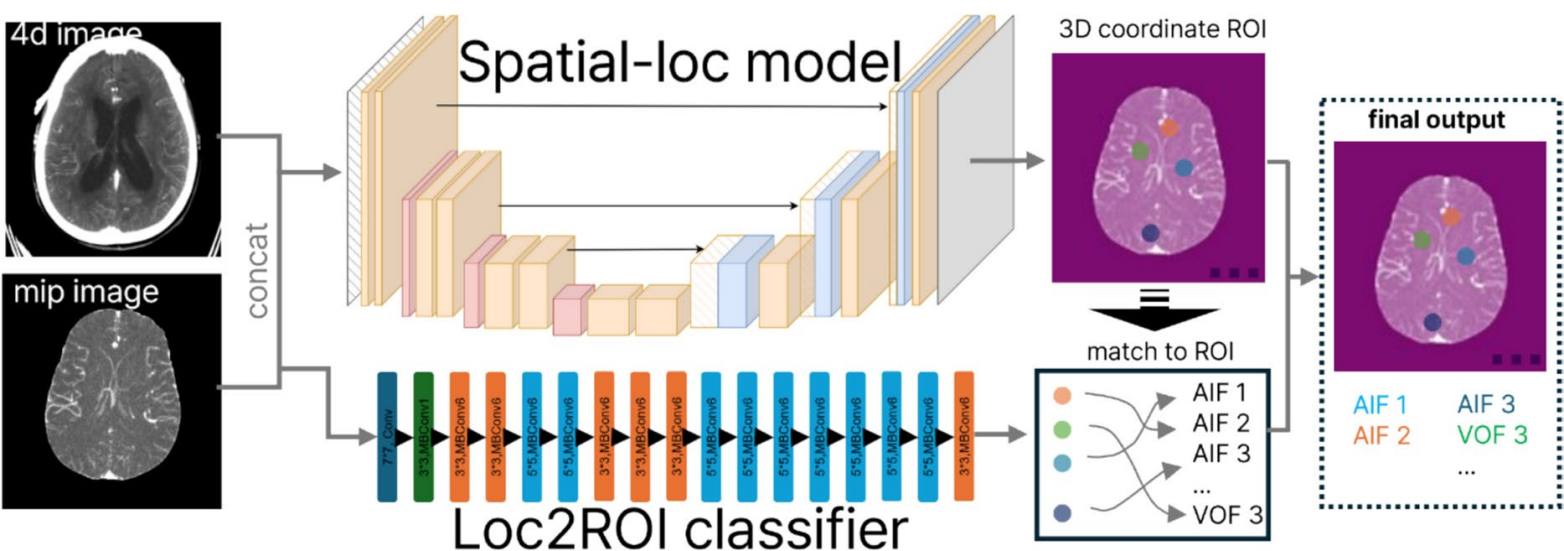
Overview of localization model. It contains two models’ input and output briefly

We referred to the CNN model used to extract the AIF/VOF curves as the localization model; its overall architecture is depicted in Fig. 2. This model comprises a U-Net-based network utilizing ResNet-50 as the encoder and an EfficientNet-b6 model serving as the classifier [17–19]. The model takes an MIP image as input, along with a 4 d image, and predicts 8 ROIs. 8 ROIs consist of five AIF and three VOF. The precise position of each candidate is determined from the selected ROIs.

Next, we extracted the intensity curves at these ROIs from the original CTP image, which consisted of 3D + T. Additional intensity curves were extracted using a grid search algorithm within an eight-voxel radius around each position. Hereafter, we refer to this method as the ROI correction. Among all the extracted intensity curves, the one with the highest area under the curve was selected for each candidate, resulting in five AIF and three VOF candidates that were optimal.

#### Validity Checking Model

We introduced an approach that enables early detection of image anomalies by analyzing the intensity curves extracted from accurately localized ROIs. Despite using the correct ROIs, the resulting intensity curves may reveal inconsistencies or artifacts that suggest underlying issues, such as image quality degradation.

**Fig. 3:**
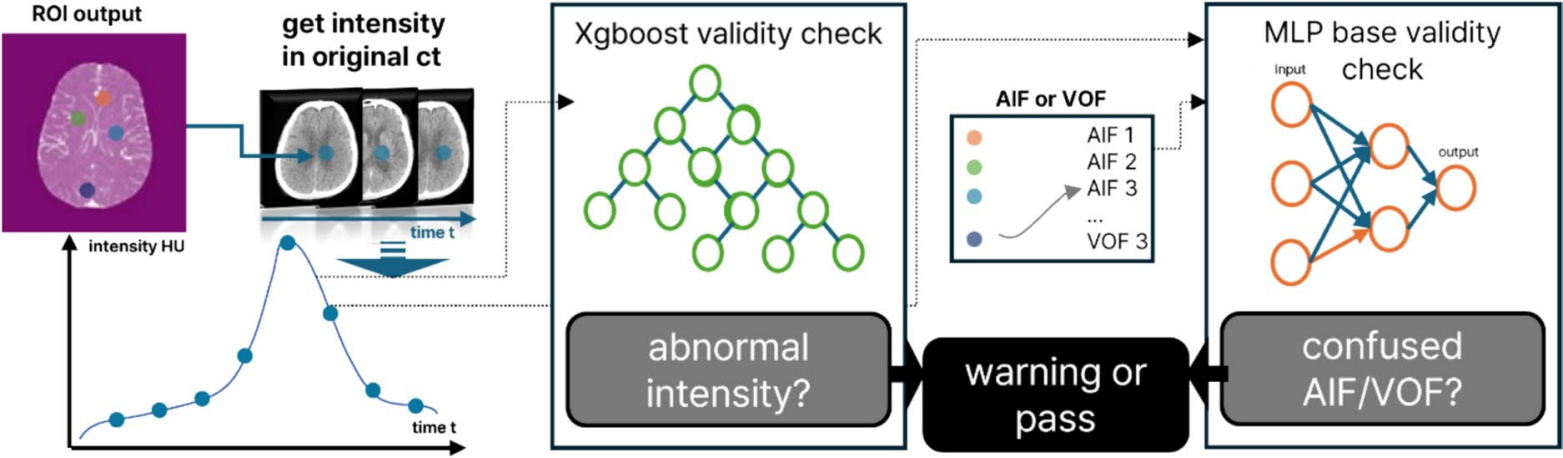
Overview of intensity based-validity checking model and landmark based-validity checking model

Figure 3 illustrates these two models. First, the intensity-based validity-checking model (IV model) consists of two XGBoost models trained on intensity curves extracted from annotated vessel locations corresponding to AIF and VOF, along with labels indicating the validity of these intensity curves [20]. Ground truth for IV model was manually annotated using rule-based methods such as sharp peak, peak Hounsfield units (HU) > 80, relative faster peak timing with narrow width (in case of AIF), no jagged or multi-peak, fast attenuation to baseline after reaching peak, having 3 ~ 12 s temporal delay between AIF and VOF [6]. Using intensity curves as training data, the models were also trained with data augmentation through Gaussian noise. This approach simulates jagged and multi-peak intensity curves, which can arise due to factors such as patient motion [6]. The IV model for AIF candidates was trained exclusively on the AIF data, whereas the IV model for VOF candidates was trained exclusively on the VOF data. Each model assesses intensity curve validity with rule-based methods, including peak Hounsfield units (HU) and shaking HU.

Next, the landmark-based validity-checking model (LV model) is trained using the same intensity curves as the IV model but is designed to determine whether a given intensity curve corresponds to the VOF. Ground truth for LV model was constructed from vessel annotation categories. VOF generally exhibits a later peak HU than AIF. Since the ROIs predicted as AIF/VOF by the localization model may not align with actual vessels, both ROIs could display the peak HU simultaneously. Without this step, there is a risk of calculating the perfusion map using incorrect pairs such as VOF with VOF or AIF with AIF. To address this issue, we trained five models: Logistic Regression, Random Forest, AdaBoost, Multi-Layer Perceptron (MLP), and XGBoost, and selected the MLP that demonstrated the best performance [21, 22].

After selecting the validated pairs of AIF/VOF ROIs using these two models, we identified the optimal intensity curves using a rule-based method and used them to calculate the perfusion map using bcSVD. Rules to verify the intensity curves contain whether the curve has a single peak, faster peak timing (in case of AIF), peak height, and full-width half maximum. If validated pairs could not be found for the given data using these two models, a warning was displayed. As outlined in the “Dataset” section, the data were excluded separately from Validation 2.

#### Block-Circulant Singular Value Decomposition

In perfusion analysis, the process of deconvolution using two functions is the most crucial, and singular value decomposition (SVD) is the most effective method for performing this task [23, 24]. Briefly, with a given AIF and unknown residual function (Fr), the density function from each voxel (Fv) can be calculated as following Eq. (1):

1 $$Fv\left(t\right)=CBF\left(AIF\left(t\right)\otimes Fr\left(t\right)\right)=CBF\int_0^tAIF\left(\mu\right)Fr\left(t-\mu\right)d\mu$$

where $t$ is total time and $\otimes$ represents the convolution. Given the function $Fv\left(t\right)$ and $AIF\left(t\right)$ with a total length of N, this relationship can be expressed discretely as (2):

2 $$Fv\left(t_j\right)=\triangle t\bullet CBF\sum\nolimits_{i=0}^jAIF\left(t_i\right)Fr\left(t_j-t_i\right)$$

where $j$ represents each timepoint (0,1,2…, N-2, N-1). To obtain the $Fr$ using this formula, the SVD can be applied on the matrix (3):

3 $$\begin{bmatrix}\begin{array}{c}F_v\left(t_0\right)\\F_v\left(t_1\right)\\\dots\end{array}\\F_v\left(t_{N-1}\right)\\F_v\left(t_N\right)\end{bmatrix}=\Delta t\begin{bmatrix}\begin{array}{cc}AIF\left(t_0\right)&0\end{array}\begin{array}{cc}\dots&0\end{array}\\\begin{array}{cc}AIF(t_1)&AIF(t_0)\end{array}\begin{array}{cc}\dots&0\end{array}\\\begin{array}{cc}AIF(t_2)&AIF(t_1)\end{array}\begin{array}{cc}\dots&0\end{array}\\\begin{array}{cc}\dots&\dots\end{array}\begin{array}{cc}\dots&\dots\end{array}\\\begin{array}{cc}AIF(t_{N-1})&AIF(t_{N-2})\end{array}\begin{array}{cc}\dots&AIF(t_0)\end{array}\end{bmatrix}\times\begin{bmatrix}\begin{array}{c}F_r\left(t_0\right)\\F_r\left(t_1\right)\\\dots\end{array}\\F_r\left(t_{N-1}\right)\\F_r\left(t_N\right)\end{bmatrix}\bullet CBF$$

then this Eq. (3) can be simplified as,

4 $$V=Ar$$

where $V$ is N × 1 matrix related to $Fv$, $A$ is the N × N matrix incorporating $\Delta t$ and AIF-related terms, $r$ is the N × 1 matrix containing the product of ${F}_{r}$ and CBF. By utilizing SVD, the matrix A can be decomposed into $A=US{V}^{t}$, and the inverse of this can be multiplied, $r = {A}^{-1}V$ so we can calculate the residual function.

However, SVD methods may introduce errors due to variations in contrast agent arrival times across voxel locations, leading to inaccuracies perfusion parameters estimation. To mitigate these errors, the bcSVD method was proposed, incorporating a block-circulant matrix to better account for the varying arrival times of the contrast agent [25]. By converting the AIF-related matrix into a circulant matrix, this approach improves the accuracy of perfusion analysis by reducing sensitivity to delay and dispersion effects. Assuming that the elements of matrix A are ${a}_{ij}$, the new elements of block circulant matrix ${\overline{a}}_{ij}$ are given by the following Eq. (5):

5 $$\overline{a_{ij}}=\left\{\begin{array}{c}a_{ij},j\leq i\,\\a_{2N-j+o,0}j>i\end{array}\right.$$

### Evaluation Metric

To evaluate the performance of the localization model, we used the mean Euclidean distance difference [26]. The ground truth (GT) for the ROI localization performance is set to the ROI location manually annotated by expert radiologists as described in the “Labeling for ROI Localization” section. The ground truth (GT) was set to the ROI location manually annotated by expert radiologists and those obtained from commercial software, as described in the “Labeling for ROI Localization” section. A lower distance difference indicates accurate localization of the vessel. Equation (6) represents the 2D Euclidean distance.

6 $$\begin{array}{c}d\left(p,q\right)=\sqrt{\left(p_1-q_1\right)^2+\left(p_2-q_2\right)^2}\end{array}$$

where $p$ and $q$ are the coordinates determined by ${p}_{1}, {p}_{2}$ and ${q}_{1},{q}_{2}$, respectively. $d$ is the Euclidean distance.

To evaluate the volume prediction performance of the proposed method, we utilized concordance correlation coefficient score (CCC) [27]. The GT for the volume prediction performance is set to the volume values from RapidAI. Since volume prediction shows whole pipeline’s performance, we use RapidAI’s automated volume prediction result as GT since proposed method also targets automated volume prediction. The CCC score measures the agreement with the given result. The CCC is given by Eq. (7):

7 $$\begin{array}{c}CCC=\frac{2\cdot PCC\cdot\sigma x\cdot\sigma y}{\sigma x^2+\sigma y^2+\left(\overline x-\overline y\right)^2}\end{array}$$

where $\overline{x}$ is the mean of $x$, $\overline{y}$ is the mean of $y$, $\sigma x$ is the standard deviation of $x$, $\sigma y$ is the standard deviation of $y$, and $PCC$ is the Pearson’s correlation coefficient between volumes from proposed method and RapidAI. We also conducted statistical analysis to see whether the addition of each module enhances CCC using 1000 bootstrap samples per method to estimate the CCC values [28]. We then applied linear regression and linear mixed model to evaluate module count and model agreement as well [29].

To evaluate the input data assessment ability of the proposed method, we used warning accuracy. Equation (8) represents the warning accuracy.

8 $$\begin{array}{c}WarningAccuracy=\frac{\text{Correct Warnings}}{\left(\text{Correct Warnings}+\text{Incorrect Warnings}\right)}\end{array}$$

Correct warnings are the number of cases in which each method issued a warning regarding inappropriate CTP images. Incorrect warnings are the number of cases in which each method issued warnings to the appropriate CTP images. For statistical comparison, we conducted two complimentary analysis, proportion z-test and generalized estimating equations (GEE) to see which method has higher accuracy than the opposition [30]. More detailed information is provided in the “Comparison of Input Data Assessment Between RapidAI and Proposed Method” section.

To compare volume prediction performance based on DWI, we used mean absolute error (MAE). Equation (9) represents the MAE.

9 $$\begin{array}{c}MAE=\frac1n\sum_{i=1}^n\vert y_i-\widehat{y_i}\vert\end{array}$$

where $n$ is the number of samples, ${y}_{i}$ is the ground truth, and $\widehat{{y}_{i}}$ is the predicted value.

### Implementation

Model training was conducted on a system equipped with four NVIDIA RTX 4090 GPUs, Intel Xeon Gold 6326 CPU, and 768 GB RAM running Ubuntu 20.04. Training hyperparameter for localization model is as follows: no pretrained weight, 5000 training epochs, mean squared error loss function, Adam optimizer with learning rate of 0.00008 and StepLR scheduler. Hyperparameter tuning of machine learning models was done using Optuna(https://optuna.org/), which enables us to optimize adequate parameters faster than grid or random search using Tree-structured Parzen Estimator. With XGBoost package (https://xgboost.readthedocs.io/en/stable/), training hyperparameters for the IV model were tested among these options: the number of estimators, max depths, learning rate, gamma, maximum delta step, and class balancing weights. Finally, the chosen hyperparameter is as follows: 100 estimators, 78 depths, a learning rate of 0.07, 5 gamma, 10 maximum delta steps, and 0.1118 class balancing weights. On the other hand, training hyperparameters for the LV model were tested with MLP Classifier of the scikit-learn package. This model was tested among various options, including activation function, solver, alpha, initial learning rate, learning rate decay, and max iteration. The best hyperparameters chosen are logistic activation, Adam optimization solver, 0.000139 alpha, 0.000272 initial learning rate, decreasing learning rate, and 4000 max iterations. Other hyperparameters that are not reported here were set as default values.

## Results

**Fig. 4:**
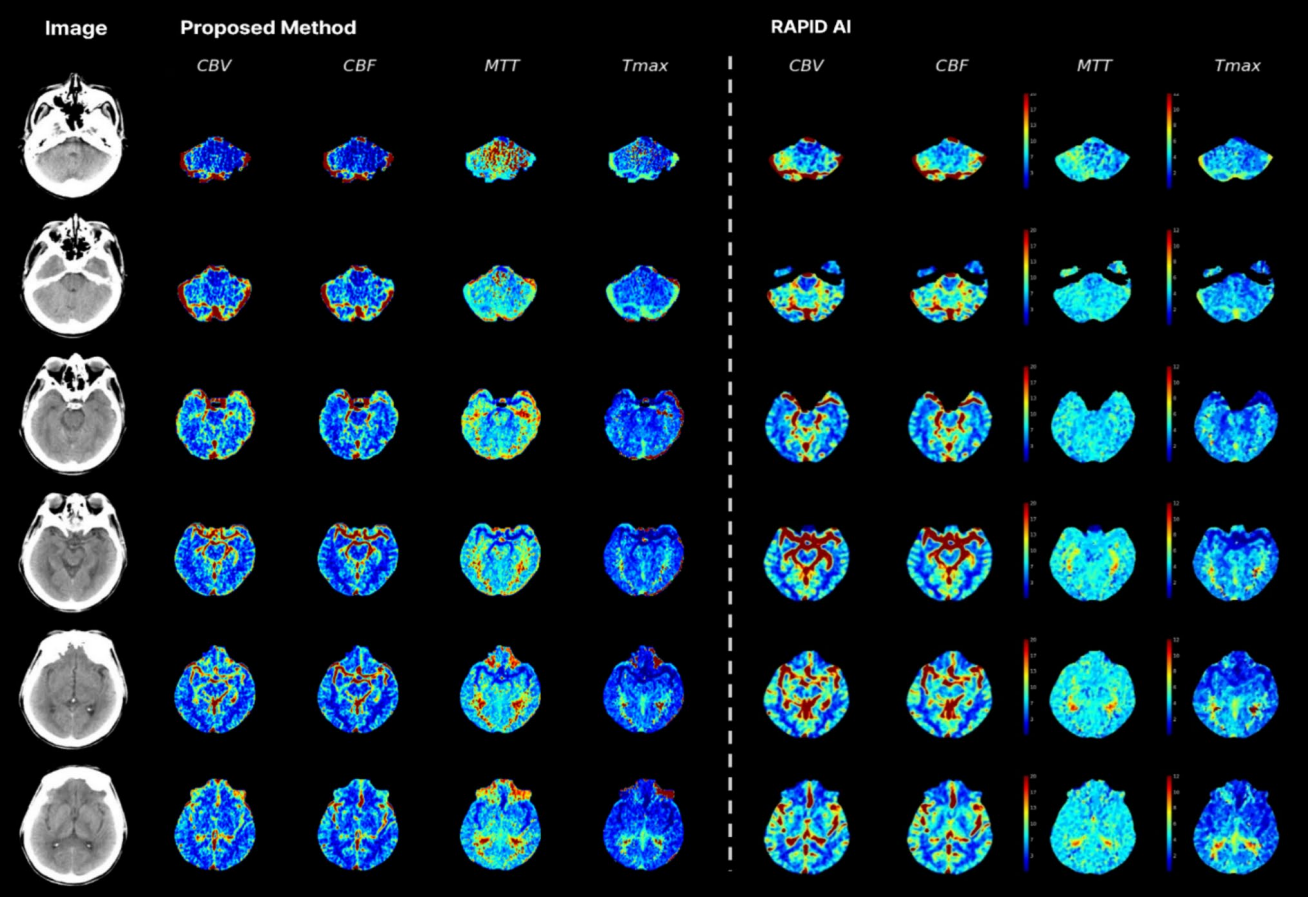
Overview of cumulative map generated by proposed method and RAPID

Figure 4 illustrates an overview of the cumulative map generated by the proposed method and RapidAI on the same patient. For each method, we selected the slice that most closely corresponds to the reference for visualization.. From the MIP image on the left, the proposed method generates perfusion maps comprising CBV, CBF, MTT, and Tmax. Using the CBF and Tmax map, Volume F and Volume T were predicted. Similarly, RapidAI generates the same four maps for the given CTP image.

The “Results of Localization Model” and “Results of ROI Correction and Intensity and Landmark-Based Validity Checking Models” sections detail the performance and application outcomes of each module comprising the proposed method. In the “Comparison of Volume Similarity Between RapidAI and the Proposed Method” section, we examine how the modules introduced in the “Results of Localization Model” and “Results of ROI Correction and Intensity and Landmark-Based Validity Checking Models” sections affect the prediction of Volume F and Volume T by comparing the results with and without their application. For the “Results of Localization Model,” “Results of ROI Correction and Intensity and Landmark-Based Validity Checking Models,” and “Comparison of Volume Similarity Between RapidAI and the Proposed Method” sections, we assume RapidAI outputs as the GT. As described in the “Dataset” section, we utilized the Validation 1 dataset for these analyses, and in the “Comparison of Input Data Assessment Between RapidAI and Proposed Method” section, we compared the proposed method with RapidAI from an input data assessment ability perspective. Here, we used expert assessments as the GT to evaluate both the proposed method and RapidAI. As explained in the “Dataset” section, the Validation 2 dataset was employed for this evaluation. In the “Comparison of Volume Difference Between RapidAI and the Proposed Method to DWI-Based Infarction Area” section, we compare proposed method with RapidAI in terms of volume similarity using DWI as GT.

### Results of Localization Model

Table 2 presents the mean Euclidean distances of the localization models for each AIF and VOF landmark. The overall mean Euclidean distance difference across all landmarks is 4.63 $\pm$ 2.00 mm, indicating that the predicted locations are close to the ground truth positions annotated by expert radiologists. Notably, for AIF_3, the average error was less than 2 mm, demonstrating strong localization performance. However, the Euclidean distance for VOF were generally larger than those for the AIF locations.

**Table 2:** Localization model test result. Differences are in millimeters

Landmark	Difference in mean distance (95% CI)
AIF_0	4.54 (4.27–4.81)
AIF_1	4.85 (4.53–5.17)
AIF_2	2.65 (2.42–2.88)
AIF_3	1.95 (1.66–2.24)
AIF_4	3.18 (2.84–3.52)
VOF_0	5.27 (4.90–5.64)
VOF_1	7.37 (6.98–7.76)
VOF_2	7.25 (6.92–7.58)

### Results of ROI Correction and Intensity and Landmark-Based Validity Checking Models

**Fig. 5:**
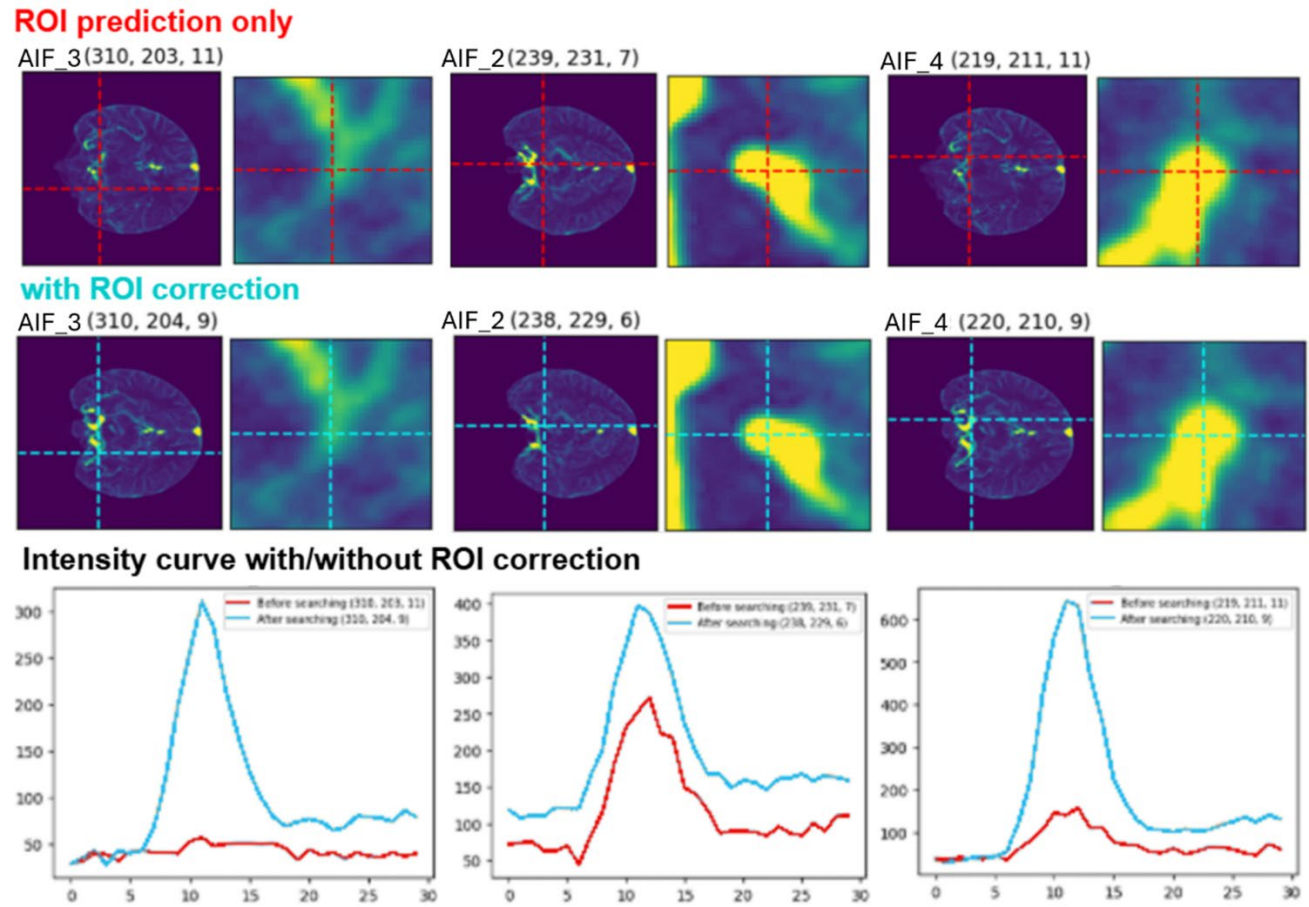
Example of how ROI correction applied. Red line shows original intensity curve, and blue line shows intensity curve after ROI correction applied

Figure 5 illustrates an example of an ROI corrected using ROI correction and the resulting changes in the intensity curve. In the upper left of Fig. 5, for AIF_3, the corrected location after ROI correction differs by one voxel on the y-axis and two voxels on the z-axis. This coordinate difference was immediately visualized to the right, showing minimal differences that were barely noticeable to the naked eye. However, when examining the AIF_3 intensity curve, it was evident that the intensity curve, which previously exhibited almost no variation in HU and was nearly flat, displayed a normal intensity curve after location correction through ROI correction. This demonstrates that applying ROI correction can be highly effective in certain cases.

**Fig. 6:**
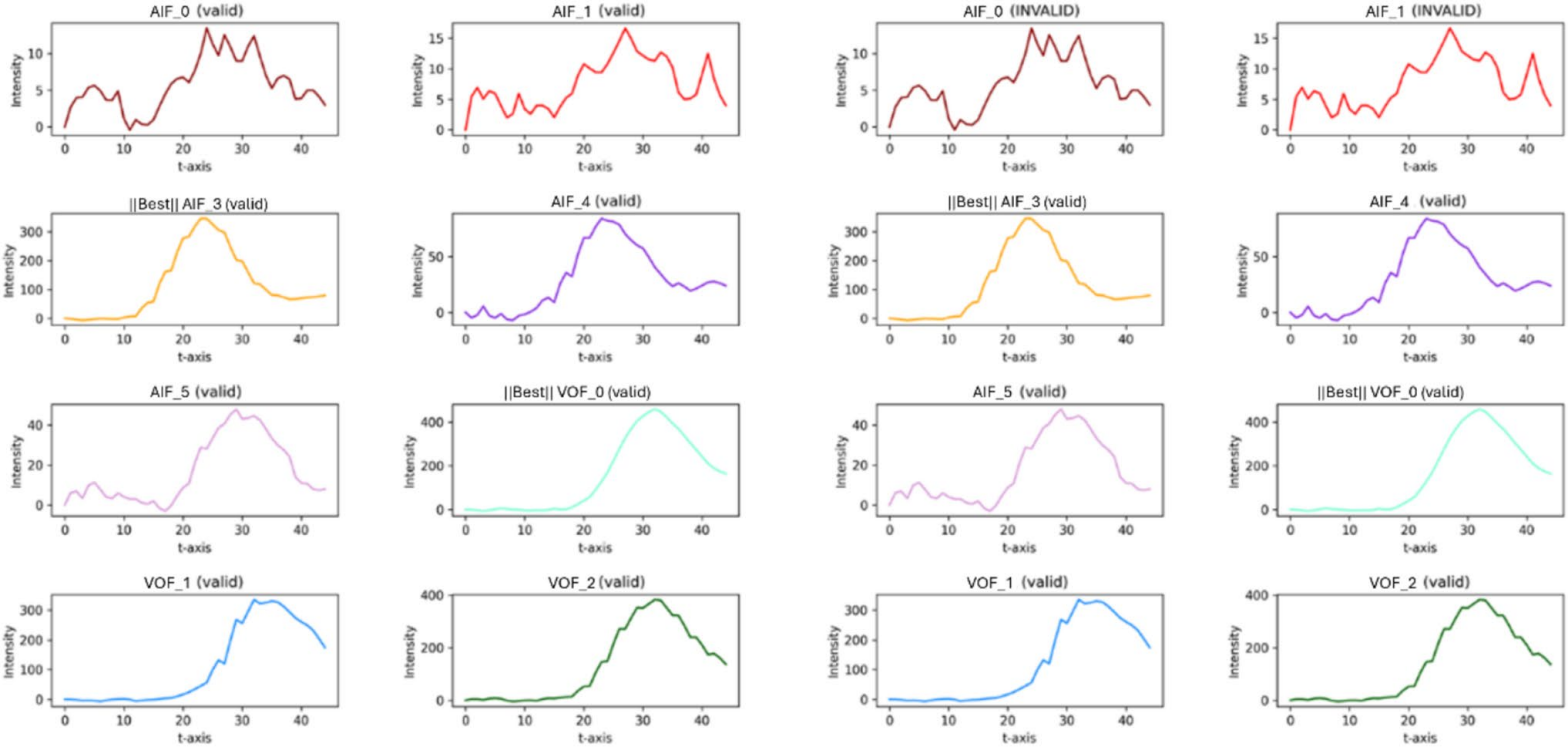
Example of IV model result. On the left side, it shows the original result and on the right side, it shows the IV model applied result. Each validity for given intensity curve is shown on top of each intensity curve

Figure 6 depicts the results of the intensity validation judged by the rule-based method and IV model. In the cases of AIF_0 and AIF_1 in Fig. 6, the intensity curves are irregular and difficult to consider as normal. However, the rule-based method on the left, which judges the validity based on factors such as the maximum HU corresponding to the bone or the slope of the intensity curve, deems them valid. In contrast, the IV model on the right classifies these intensity curves as invalid.

**Fig. 7:**
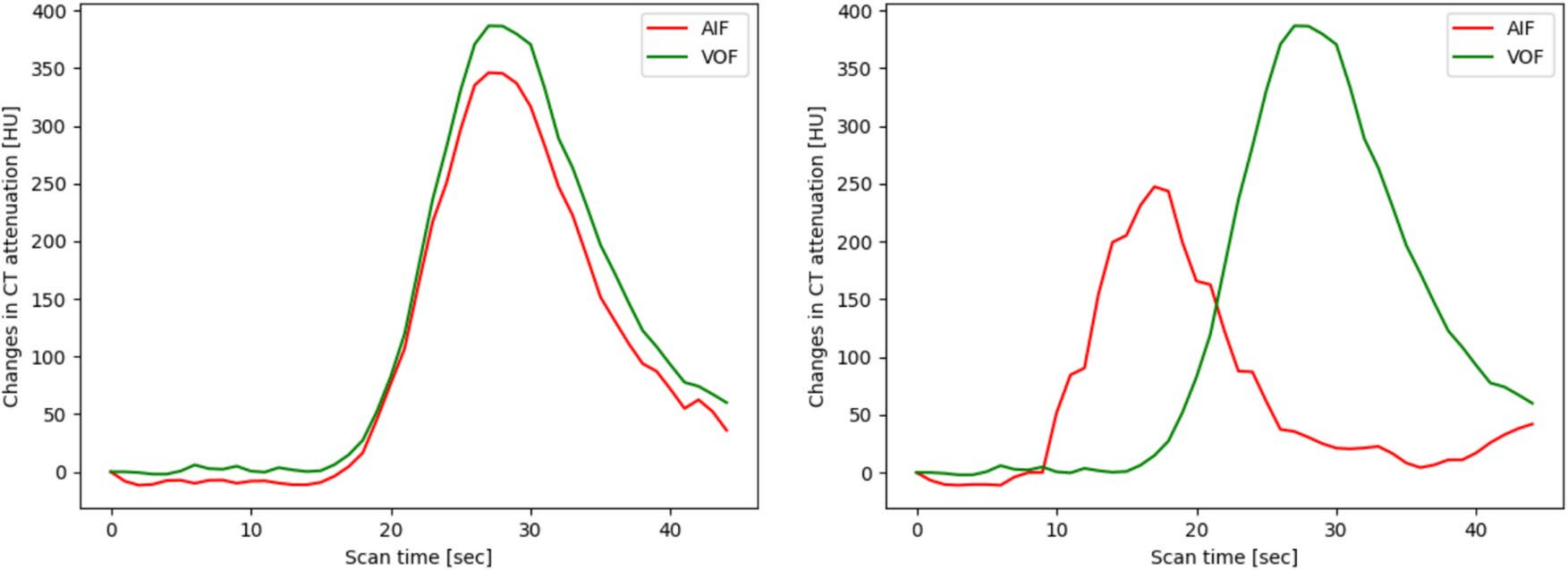
Example of how LV model affects AIF/VOF relationship in intensity curve. On the left side, it shows the relationship between AIF/VOF without LV model and On the right side, it shows the relationship between AIF/VOF with FV model. On the right side, the VOF’s peak HU is behind the AIF’s peak HU

Figure 7 shows an example of the application of the LV model. The left of Fig. 7 shows the AIF and VOF curves before applying the LV model. Here, AIF and VOF reached their peak HU at almost the same time. Considering that the peak HU of the VOF typically appears after that of the AIF, the AIF shown in the figure more closely resembles that of a VOF. The right side of Fig. 7 displays the LV model-corrected curves. Unlike the left side, where the AIF and VOF curves overlapped, the right side shows that the peak HU of the AIF appears before that of the VOF. This adjustment results in an AIF and VOF pair that aligns more closely with the typical physiological relationship between them, which is expected to contribute to improved volume prediction.

### Comparison of Volume Similarity Between RapidAI and the Proposed Method

Table 3 presents the experimental results comparing the application of ROI correction and the intensity curve validation process using the ML models. The CCC score was calculated using the volume values from the RapidAI as the GT and represents the average across all subjects. The localization model refers to the volume prediction results obtained using only the localization model. ROI correction refers to the results obtained after introducing a method to obtain better intensity curves within an 8-voxel radius around the locations identified by the localization model. The IV and LV models refer to the results obtained after introducing a method to validate the given intensity curves. With the application of ROI correction, a slight increase in the CCC score for each volume was observed, highlighting ROI correction’s role in improving performance. When applying the IV and LV Models separately with ROI correction, the performance improvement was more pronounced with the IV Model than with the LV Model for Volume T. The highest performance was achieved when each module was applied, suggesting that each module contributed to performance improvement. Additionally, as every module was applied, the CCC score for Volume T increased significantly, approaching that of Volume F. In the linear regression analyses conducted separately for the two regions (Volume F and Volume T), we observed a statistically significant positive association between the number of modules and CCC. Specifically, the estimated effect size was 0.0012 (*p* < 0.001) for Volume F and 0.0365 (*p* < 0.001) for Volume T, indicating that methods with more modules tended to achieve higher CCC values consistently across both regions.

**Table 3:** CCC score of proposed method using RapidAI as ground truth

Method (CCC score)	Volume F	Volume T
LOC	0.898 (CI, 0.858–0.930)	0.715 (CI, 0.546–0.874)
LOC + COR	0.902 (CI, 0.864–0.933)	0.722 (CI, 0.550–0.880)
LOC + COR + IV	0.897 (CI, 0.861–0.934)	0.869 (CI, 0.828–0.905)
LOC + COR + LV	0.901 (CI, 0.859–0.934)	0.764 (CI, 0.622–0.895)
LOC + COR + IV + LV	**0.905 (CI, 0.862**–**0.939)**	**0.879 (CI, 0.836**–**0.914)**

*LOC* localization model, *COR* ROI correction, *IV* IV model, *LV* LV model, *CI* confidence interval

The numbers in bold indicate the highest scores

Recognizing the repeated-measures structure of the data—where CCC values from Volume F and Volume T are correlated within the same method—we further employed a linear mixed-effects model with region specified as a random effect. This approach confirmed a significant positive effect of feature number on CCC (Fixed Effect Estimate = 0.0189, *p* < 0.001), accounting for within-model correlation and variability between regions.

These results strongly support that proposed method with more modules yield better agreement. Volume distribution on proposed method’s LOC + COR + IV + LV and RapidAI are as follows. Volume F proposed method mean (standard deviation, std), median (interquartile range, IQR): 25.6768 (± 44.4006), 8 (3–23), Volume F RapidAI mean (std), median (IQR): 21.0414 (± 42.6733), 0 (0–17), Volume T proposed method mean (std), median, (IQR): 76.6795 (± 104.3901), 39 (7–104), Volume T RapidAI mean (std), median (IQR): 68.7430 (± 86.3928), 38 (6–93).

### Comparison of Input Data Assessment Between RapidAI and Proposed Method

The RapidAI software specifies that appropriate CTP images for analysis should be acquired using specific imaging protocols, such as a total scan time of 60–70 s and injection of 40 ml of contrast agent at a rate of 4–6 ml/s [6]. Even though explicit guidance has been provided, several studies have emphasized the importance of caution when interpreting automated CT perfusion software analysis reports due to technical and clinical pitfalls, such as patient motion, poor contrast bolus, and volume measurement errors, which could result in preprocessing failure [7, 31–33]. Obviously, when these pitfalls are identified by automated CT perfusion software before generating an analysis report, it can prevent the presentation of misleading information to the radiologist. However, some studies found instances where automated CT perfusion software failed to detect these pitfalls, resulting in incorrect analysis reports that required correction from a radiologist [8, 9]. This highlights the need for more reliable pitfall detection capabilities in predicting brain volume affected by AIS to ensure accurate and consistent analysis results.

Given this critical need, we defined input data assessment ability in our study as the method’s ability to as follows:

(A) Correctly identify inappropriate CTP images by issuing a warning.

In this context, when either the proposed method or the RapidAI issued a warning, the image was considered inappropriate (Case A). Thus, we can measure the accuracy with which the proposed method identifies inappropriate CTP images.

To evaluate the input data assessment ability, we collected CTP images where RapidAI and the proposed method issued warnings, which is referred to as Validation 2 in the “Dataset” section. Subsequently, an expert radiologist independently reviewed the appropriateness of the collected CTP images for predicting the affected brain volume. These were categorized into three groups: (a) not appropriate, (b) caution required when used, and (c) appropriate. In this case, of the 81 CTP images from Validation 2, 26 CTP images fell into category (a), 34 into (b), and 21 into (c). Since case (c) was not applicable to the input data assessment ability definition above, it was excluded, and only cases (a) and (b) served as the GT for evaluating the input data assessment ability. Specifically, CTP images belonging to case (b) were considered usable for analysis, though expert radiologists noted a high likelihood of generating inaccurate volume prediction results. In cases (a) and (b), both the proposed method and RapidAI were allowed to perform analyses using the same source CTP image. We then collected volume prediction reports to examine the number of warnings issued by each method to calculate the accuracy of the issued warnings.

Table 4 presents the number of issued warnings from the proposed method and the RapidAI on the CTP image belonging to Cases (a) and (b) with its accuracy. Out of 60 CTP images, the proposed method issued incorrect warnings to 12 CTP images, while RapidAI issued 23 incorrect warnings. The proposed method achieved an accuracy of 0.8, whereas RapidAI achieved an accuracy of 0.62. Based on the prior definition of input data assessment ability, it can be concluded that the proposed method identified inadequate input images. Additionally, a more accurate warning implies a more reliable volume prediction, which can facilitate a more informed decision-making process for radiologists. In Proportion z-test, we compared the accuracy rates (proportions of correct predictions) between two methods. The observed accuracy was 0.8 for proposed method and 0.617 for RAPIDAI. The z-test yielded a statistic of 2.21 with a *p*-value of 0.014. As a result, the null hypothesis, there is no difference in accuracy between the proposed method and RapidAI, was rejected, indicating that proposed method has significantly higher accuracy than RapidAI. This tendency was also supported by the result of GEE. Fitting a logistic regression model with each method as a predictor, the model estimated a log-odds increase of 0.911 for correct classification when using the proposed method compared to RAPIDAI (*p* = 0.064), suggesting a trend toward improved classification accuracy. Although the result did not reach the conventional level of statistical significance (*p* < 0.05), it indicates a potential performance advantage of proposed method at the 0.10 significance level.

**Table 4:** Comparison of accuracy between A and B for warning cases

Method	Correct warnings	Incorrect warnings	Accuracy
Proposed method	**48**	**12**	**0.80**
RapidAI	37	23	0.62

The numbers in bold indicate the highest scores

**Fig. 8:**
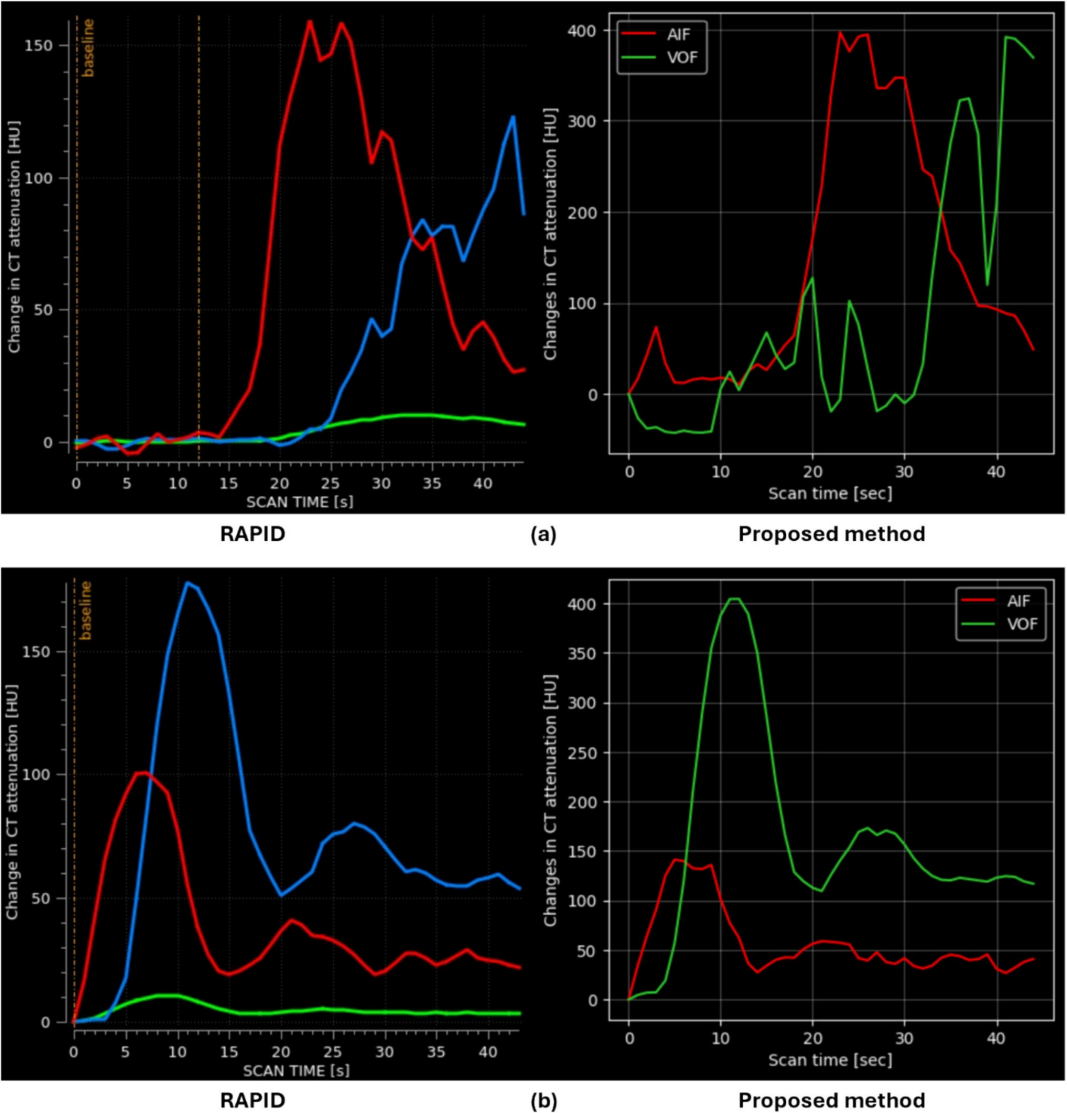
Example of CTP image from case (**a**) and (**b**)

Figure 8 displays the CT attenuation graphs for the CTP images from cases (a) and (b), where warnings were issued by the proposed method but not by RapidAI. In Fig. 8a, both CT attenuation graph shapes exhibit similar characteristics: multiple peaks in AIF and VOF, along with fluctuating HU values. These features are easily noticeable and highlight the need for correction, which can be manually performed by a radiologist through replacing the ROI on the CTP image. Failure to detect these inaccuracies during the validation process may lead to errors in volume prediction, potentially affecting the treatment process. This suggests that the proposed method may be more sensitive in detecting inappropriate CTP images, thereby preventing potential inaccuracies in the analysis.

In Fig. 8b, both CT attenuation graph shapes exhibit similar characteristics. However, the proposed method demonstrated higher AIF/VOF peak HU than that of RapidAI. This implies that the IV models are successfully integrated into the proposed method, as they can distinguish the validity of AIF/VOF beyond rule-based criteria, such as peak HU. Furthermore, the onset of the increasing timing occurs more rapidly than the CTP image acquisition parameters, which are approximately 5–10 s from the pre-contrast baseline [6]. These findings indicate that our hybrid deep and ML model can accurately distinguish between appropriate and inappropriate CTP images, potentially reducing unnecessary warnings.

### Comparison of Volume Difference Between RapidAI and the Proposed Method to DWI-Based Infarction Area

To ensure a fair comparison between the predicted volumes and DWI-based ground truth, we excluded CTP-DWI pairs acquired more than 3 h apart, as infarct volumes can change significantly over time [34]. Consequently, out of 51 DWIs in Test 1, only 16 met this criterion. The infarction areas of these data were first annotated by a trained annotator, using a mask file segmented with ADC ≤ 620 × 10⁻⁶ mm^2^/s threshold. These files were then sequentially reviewed and refined by a resident radiologist and 13 years of experienced expert radiologist [35]. The infarction volume of these data are 10.737 ± 17.664 ml and their acquisition time gaps between DWI and CTP are 87.552 ± 49.961 min. Among these data, 13 of them had large vessel occlusion and 7 of them had stenosis. Table 5 summarizes the mean absolute error (MAE) of each method on these cases, using DWI as the ground truth for Volume F. The proposed method achieved a lower MAE (7.917, ± 15.203) compared to RapidAI (11.343, ± 16.943), indicating improved accuracy and robustness. The relatively high standard deviations may reflect inter-patient variability.

**Table 5:** Comparison of MAE between Volume F from each method to DWI-based infarction

Method	MAE
Proposed method	**7.917 (± 15.203)**
RapidAI	11.343 (± 16.943)

The numbers in bold indicate the highest scores

## Discussion

Our hybrid approach leverages the strengths of both deep and ML techniques. The localization model is valuable in settings where a neuroradiologist may not be readily available, ensuring that the initial analysis can proceed without expert manual intervention. Accurate AIF and VOF values are crucial for reliable perfusion analysis. ROI correction plays a key role in improving their estimation. We combined automatic ROI localization with intelligent validation processes to create a fail-safe mechanism that improves the overall accuracy. This synergy allows for more precise identification of ROIs and validation of intensity curves, leading to more accurate volume predictions.

We aimed to extract optimal intensity curves while enhancing the input data assessment ability. To achieve this, we employed a combination of ROI correction and IV and LV models, collectively referred to as the ML methods. Our results clearly demonstrate that integrating the ML methods significantly improved volume prediction accuracy. Our approach enhances the reliability of perfusion analysis even when faced with CTP images of varying qualities. This ensures high performance in real-world clinical environements, even when data quality and conditions vary.

The accuracy of AIF/VOF can be compromised by partial volume effects, which is a challenge that cannot be easily overcome even if the ROI localization model is highly accurate [36]. The ROI correction method we employed explores the intensity values around the ROI to identify locations that satisfy predefined criteria, thereby enabling a consistent level of AIF/VOF extraction even in the presence of partial volume effects.

Simultaneously, AIF/VOF possesses inherent characteristics as time-series data. Consequently, during the acquisition period of CT perfusion (CTP) imaging, there is a heightened probability of patient motion artifacts. It is well-established that patient movement adversely affects volume calculations utilizing AIF/VOF [37]. Nevertheless, the proposed method can remain relatively unaffected by this issue due to the application of ROI correction.

The improved input data assessment capability and reliability of our method have significant implications for clinical practice, particularly in terms of robustness. Consistent and accurate diagnostic tools, such as those capable of robust volume prediction, are essential for clinicians to make informed decisions, especially in time-critical conditions, such as AIS. The input data assessment ability of our model provides robust and dependable results and can improve patient outcomes by facilitating timely and appropriate interventions.

Nonetheless, these approaches have limitations. First, there is dependency on the performance of the ROI localization model, as the utility of ML methods may diminish if the ROI model’s performance is substantially improved. Subsequently, there is the potential for better methods, as alternative methods may exist that offer superior performance.

The ML methods primarily serve as a fail-safe mechanism for detecting and preventing errors when the ROI predicted by the localization model is off the vessel, the extracted intensity curve is invalid, or the predicted ROI is incorrect. This implies that as the localization model becomes more accurate, the reliance on the ML methods may decrease. However, as observed in Fig. 5, the differences between the ROI coordinates predicted by the localization model and those corrected by the ROI correction were minimal. This suggests that ROI correction is beneficial for refining the ROI when the model predicts locations close to but not precisely on the vessel.

Performance improvements reported in the “Comparison of Volume Similarity Between RapidAI and the Proposed Method” section, further support this observation. While ROI correction provided some enhancement, the ML models yielded significantly greater gains. Additionally, visual inspection of the coordinates before and after correction in Fig. 5 reveals only subtle differences, underscoring the inherent difficulty of precisely locating vessels. Therefore, while improving the performance of localization models remains a key objective, the immediate application of ML methods is beneficial and cannot be disregarded. Furthermore, the validation of AIF/VOF using this machine learning approach has been demonstrated in [38].

Moreover, the findings in the “Comparison of Input Data Assessment Between RapidAI and Proposed Method” section indicate that our hybrid DL and ML approach effectively enhances the input data assessment ability. The experimental results demonstrated that the proposed method is capable of correctly issuing warnings for an inappropriate CTP image, indicating that the proposed method possesses an appropriate level of input data assessment ability, which leads to robust volume prediction.

For instance, in Fig. 8a, for the AIF and VOF curves exhibiting multiple peaks, which is an indication of potential issues, the proposed method issued a warning. In Fig. 8b, the proposed method seems to have identified the appropriate AIF and subsequently issued a warning. These observations suggest that the proposed method offers better input-data assessment ability by accurately detecting and addressing potential problems. Consequently, in real-world scenarios, the proposed method is expected to produce more consistent and accurate results across CTP images of varying qualities, making it highly robust and promising for clinical applications.

The ML methods employed in this study were based on intuitive and widely used approaches, ensuring fast operation due to their simple architectures. However, tasks assigned to ML methods, such as intensity curve validation and AIF/VOF validation, could potentially be implemented using alternative machine learning techniques. This suggests that, regardless of improvements in the localization model’s performance, the accuracy of volume prediction could be further enhanced with the development and application of more advanced ML methods.

Comparing CTP map and DWI is useful to assess how well CTP maps correspond to the actual infarct cores identified on DWI, i.e., whether CTP can be used as a surrogate marker for DWI [34]. As shown in Table 5, the proposed method showed smaller MAE than the other method. Nonetheless, there were only 16 CTP-DWI pairs which were taken less than 3 h apart. Therefore, these limitations should be taken into consideration when interpreting the findings.

## Conclusions

This study demonstrated that our proposed hybrid DL and ML model effectively predicts AIS-affected brain volume, improving the accuracy and reliability of perfusion analysis. The integration of ML-based validation methods enhanced input data assessment, leading to more robust volume predictions even in cases with variable image quality. Our results indicate that the proposed method may offer a practical and accurate tool for assisting clinicians in stroke diagnosis and treatment planning.

In future work, we aim to further improve the localization model and explore additional ML techniques to enhance the performance. Validating our method across diverse datasets and comparing it with other commercial software will also help establish its utility and generalizability. By continuing to focus on robustness and clinical applicability, we hope to contribute to the advancement of stroke diagnosis and treatment.

## Data Availability

The data and code in the current study are not publicly available because of privacy, ethical, or legal issues but are available from the corresponding author upon reasonable request.
